# Maillard reaction in vivo and its relevance to diseases: editorial and dedication

**DOI:** 10.1007/s10719-021-09996-6

**Published:** 2021-04-24

**Authors:** Motoko Takahashi, Naoyuki Taniguchi

**Affiliations:** 1 Sapporo Medical University, South-1 West-17, Chuo-ku, Sapporo, 0608556 Japan; 2 Osaka International Cancer Institute, 3-1-69, Otemae, Chuo-ku, Osaka, 541-8567 Japan

**“Vincent M. Monnier, MD.**

**Professor of Case Western Reserve University**

**“A living legend, a leader of the field of the Maillard Reaction”**

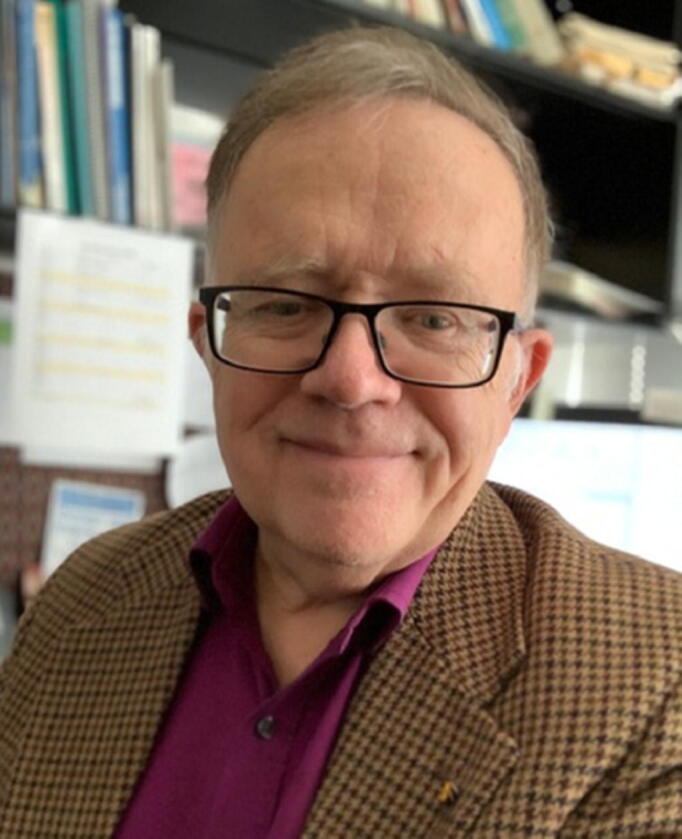



Photo: Dr. Vincent Monnier with Dr. Paul Thornalley, Dr. Naoyuki Taniguchi and his colleagues in Osaka University Medical School when he joined the IMARS meeting in 2001

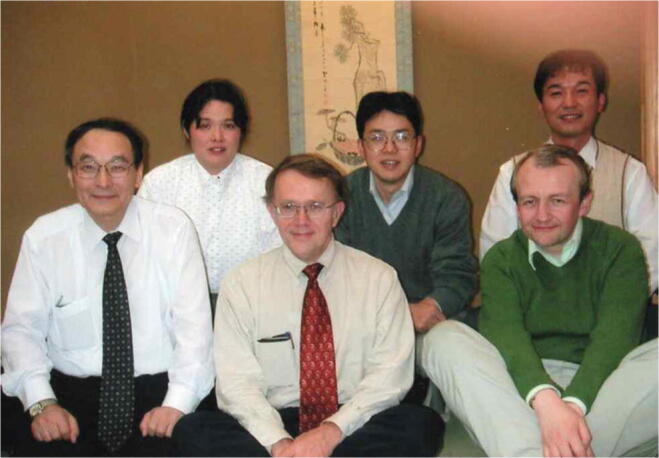



## Editorial and dedication

This document is dedicated to a living legend, a leader of the field of the Maillard Reaction, Vincent M. Monnier MD. Since he began his carrier, he has been in the center of the field and has dedicated himself to further innovations in this area of research. He was one of the original organizers of the International Maillard Reaction Society (IMARS) and continues to contribute to its ongoing activities. During his career, numerous postdoctoral researchers have passed through his laboratory, and many of them have continued to contribute to progress in the field of the Maillard Reaction as well as to the scientific community in general. He remains an active researcher in the field of Maillard chemistry and at the forefront of the field. We look back his achievements below.

Dr. Vincent Monnier was born in Switzerland, to a family of scientists. He obtained his medical degree and diploma in chemistry from the University of Basel. From 1977 to 82, he did postdoctoral research in Anthony Cerami’s lab at Rockefeller University (New York, NY). In 1982, he was appointed to the faculty of the Institute of Pathology, Case Western Reserve University, and since then, he has continued his research on in vivo aspects of Maillard Reaction, including studies with the Diabetes Control and Complications Trial (DCCT) group. He was awarded the Paul E. Lacy Award in 1990 and the Nathan Shock Award (National Institute of Aging) in 1996, and became an elected Fellow of the American Association for the Advancement of Science in 2015.

To overview Dr. Monnier’s research, we review some aspects of the history of Maillard Reaction research. The Maillard Reaction, which involves a reaction between monosaccharides and amino groups, was originally described by Maillard, in 1912 [1]. Maillard proposed that this reaction is related to the browning of food during cooking or during long-term storage, and also commented on the possibility that the reaction could occur in other circumstances such as in living cells or in soil, which subsequently turned out to be the case. Dr. Monnier published a monumental paper, “Nonenzymatic browning in vivo: possible process for aging of long-lived proteins” in *Science* in 1981 with Dr. Cerami, which proposed that the Maillard Reaction is intimately involved in the aging process [2]. He published another monumental paper, “Relation between complications of type I diabetes mellitus and collagen-linked fluorescence.” in the *New England Journal of Medicine* in 1986, which described the nature of the association of Maillard Reaction and diabetic complications in Type 1 diabetes [3]. These papers formed the basis of for new era of research regarding the Maillard Reaction, which pointed out the significance of the reaction in the human body. The in vivo Maillard Reaction is now generally referred to glycation, and the relevance of glycation to human diseases has attracted considerable interest in the international community of scholars.

One of Dr. Monnier’s achievements is the elucidation of the molecular mechanisms by which glycation and glycoxidation leads to impaired protein function. The glycation reaction is extremely complicated, and he has been examining the precise pathways and determining the structures the glycation products, including pentosidine [4–6], LW-1 [7–9], and triosidines [10]. He also developed methods for measuring in vivo glycation [11–13].

Dr. Monnier also examined the importance of glycation in aging and in age-related diseases, including diabetes, cataract formation and renal diseases, revealing that glycation theory explains the hyperglycemic memory in the pathology of diabetic complications [14–18]. His research has placed particular emphasis on the formation of collagen crosslinks which increase with age and are responsible for the progressive stiffening of vessels. Collaboration with the DCCT group revealed the association between AGEs derived from skin collagen and the progression of complications in Type I diabetes during the follow-up phase in a study of the Epidemiology of diabetes Interventions and Complications (EDIC). He has shown that glycation products reflect cumulative glycemia over a period of years, and are associated with the severity of micro- and macrovascular disease at various time-points during EDIC [19–23].

Dr. Monnier has also attempted to develop methods to slow down the speed of glycation. His search for soil microorganisms that are capable of digesting Amadori-products resulted in his discovery of FAD-containing fructosyl-amine oxidases [24, 25], and membrane-bound 1-deoxyfructosyl alkyl amino acid oxidase [26–28]. Fructosyl amine oxidases function to cleave the ketoamine bond of Amadori products to produce hydrogen peroxides and glucosone, whereas 1-deoxyfructosyl alkyl amino acid oxidase cleaves the alkylamine bond to produce fructosamine.

Since his research remains ongoing, it is not possible to completely summarize his work, but it is possible for us to review his contributions to the research community. As mentioned above, he has been involved in the advanced training of numerous researchers, including many post-doctoral fellows from Japan. Among these we include Drs. Fumitaka Hayase, Satoshi Miyata, Shinji Taneda, Motoko Takahashi, Atsushi Araki, Yoko Nishikawa, Makoto Satake. Many of those post -doctoral scientists have remained in the field of Maillard Reaction and continue to contribute to the community, including Drs. David Sell, Ram Nagaraj, Monika Pischetsrieder, Marcus Glomb, Manuel Portero-Otin, Frederic Tessier, and more. Dr. Monnier’s contributions toward the community is substantial.

Here, I (Taniguchi) would like to introduce a brief history of the IMARS and the Japan Maillard Reaction Society (JMARS).

## History of IMARS

The first symposium of IMARS was held in Uddevalla, Sweden in 1979 [29], the 2nd was held in Las Vegas, Nevada USA in 1982 [30] which was organized by Drs. George Walter (University of Oklahoma State University) and Milton Feather (University of Missouri). The 3rd meeting was organized by Dr. Masamichi Kato (University of Tokyo) and was held at Hakone in Japan in 1985 [31]. The focus of these three meetings was on multidisciplinary fields, covering chemistry, food science, analytical methods, nutrition, toxicology and related areas. The number of scientists interested in medical topics related to the Maillard Reaction have increased dramatically since the 4th meeting which was held in Lausanne, Switzerland in 1989 [32].

This year, the 19th IMARS will be held in Qatar with Dr. Paul Thornalley serving as the host. Dr. Thornalley is now the president of IMARS. JMARS has continued to scientifically contribute to IMARS activity. Drs. Reiko Inagi (University of Tokyo) and Ryoji Nagai (Tokai University) are actively involved in activities related to IMARS and edit the highlights of the Maillard Reaction in the IMARS web site and in international networks as well.

## History of JMARS

Dr. Vincent Monnier has made great contributions to the efforts to launch JMARS. He participated in the 3rd IMARS meeting that was held in Japan in 1985 and on the way back to the US he visited Hokkaido University, Sapporo, because he has a good friend there, Ichiro Fukushima, Professor of Physiology (Hokkaido University) who spent some time as a research fellow at a laboratory at Rockefeller University. At that time, I (Taniguchi) was an associate professor at the Cancer Institute at Hokkaido University. When Dr. Monnier gave a lecture on the campus of Hokkaido University School of Medicine, Fukushima asked me to chair the session. Many years ago, I was a visiting associate professor at the Cornell University Medical School, which is located just next to the Rockefeller University and Fukushima is also a good friend of mine. He lived in the housing at Rockefeller University, which was located on the upper east side, and I lived in an apartment complex called Sutton Terrace which is located just across the street from his apartment.

In 1988, Drs. John Baynes (University of South Carolina) and Vincent Monnier organized a meeting at NIH [33]. This meeting was the first opportunity to have a joint meeting of chemists who were working mainly on browning reactions and biochemists who were interested in the role of the Maillard Reaction in vivo. Kato and Taniguchi from Japan were invited to present lectures at this meeting. Dr. Kato gave an overview of the role of 3-deoxyglusosone as an intermediate in the Maillard Reaction in vivo and in vitro [34] and Taniguchi presented information on the glycation of Cu,Zn-Superoxide dismutase [35]. This fruitful and exciting meeting stimulated me to launch this type of meeting in Japan and on my return, I discussed this with several people including Dr. Kato, and Dr. Hidetaka Nakayama, a diabetologist at Hokkaido University. We made plans to launch a Maillard Reaction Research symposium at which the attendees would be scientists with interests in agricultural chemistry as well as in medicine, which was an unusual network at that time. In August 1989, I organized a research meeting at the Ono Pharmaceutical Company Institute in Fukui, Japan who generously kindly provided us with a meeting place. We met there and, in the evening, we traveled to a hot-spring nearby and continued our discussion. At the banquet at the hot spring, the main participants were Drs. Kato, Nakayama, Naoki Kashimura (Mie University), Seikoh Horiuchi (Kumamoto University), Seiichi Homma and Tadao Kurata (Ochanomizu University), and Harold F. Deutsch (Wisconsin University, visiting professor, Osaka University), among others. At that time, I was working on the glycation of Cu,Zn- superoxide dismutase (SOD) [36, 37]. Many JMARS members participated at the 4th meeting of IMARS which was held in Lausanne, Switzerland [32]. Of the 183 scientists who attended this meeting, 63 were JMARS members.

The second research meeting was again held at the Ono Pharmaceutical Company in Osaka and was organized by Naoyuki Taniguchi. Since the 3rd meeting at Kyoto, which was organized by Dr. Yukio Shigeta (Shiga University Medical School), many clinicians with interests in in diabetes mellitus participated in the meeting and many additional persons with interests in clinical aspects of diabetes from various locations in Japan also attended the meeting. The meetings were the 9th meeting and, in 2000, the meeting name was changed to the 10th JMARS and was organized by Dr. Toshihiko Osawa (Nagoya University).

In 2001, the 7th IMARS and 11th JMARS joint meeting at Kumamoto was organized by Drs. Seikoh Horiuchi, Taniguchi, Fumitaka Hayase (Meiji University), Tadao Kurata and Toshihiko Osawa [38], and thereafter, JMARS was held in Kyoto and was organized by Dr. Shigeta whose interests were inn diabetes mellitus. In 2015 the 12th IMARS was held in Tokyo as a joint meeting with the 25th JMARS (Dr. Teruo Miyazawa, Tohoku University). After the meeting, the special issue was published in the Glycoconjugate Journal [39]. From the year 2016 to 2020, Drs. Yuichi Kaji (Tsukuba University), Teruyuki Usui (Kagawa Nutriton University), Motoko Takahashi, Kiyotaka Nakagawa (Tohoku University) and Yasuhiko Yamamoto (Kanazawa University), respectively, organized the annual meeting of JMARS .

Finally, we wish to thank all those who contributed to this Special Issue on: Advances in Glycation: from food to human health and disease. We also wish to express our particular thanks to the Glycoconjugate Journal staff, Editor Prof. Sandro Sonnino and the production staff at Springer Co., for their help with publishing these proceedings. We finally wish to acknowledge Dr. Milton S Feather for his help with the review this Editorial.
